# Fight, flight, freeze, fawn: mapping emergency department staff stress responses to workplace violence — a mixed-methods study

**DOI:** 10.3389/fpsyt.2026.1952758

**Published:** 2026-10-06

**Authors:** Jennifer Davids, Greg de Moore, Martin Brown, Margaret Murphy

**Affiliations:** 1 Research and Education Network, Western Sydney Local Health District, Sydney, NSW, Australia; 2 School of Medicine at Western Sydney University, Sydney, NSW, Australia; 3 School of Medical Sciences in the Faculty of Medicine and Health, University of Sydney, Sydney, NSW, Australia; 4 Emergency and Critical Care Unit, North Sydney Local Health District Sydney, Sydney, NSW, Australia; 5 School of Nursing and Midwifery, University of Technology Sydney, Sydney, NSW, Australia

**Keywords:** emergency department, mental health, staff, trauma, violence

## Abstract

**Background:**

Workplace violence and aggression are escalating in emergency departments (EDs) globally, yet the self-reported psychological and behavioural responses in clinicians to such stressors remain underexplored. Trauma theory conceptualises stress responses as fight, flight, freeze, and fawn (4Fs); but how these responses manifest in ED staff before, during and after violent incidents has received limited empirical attention.

**Methods:**

This mixed-methods study analysed data collected between 2019 and 2023 across four emergency departments within one Australian local health district. Data sources included semi-structured interviews (2019; n = 20), focus groups (2021; n = 30 participants), a cross-sectional survey (2023; n = 61), and follow-up interviews (2023; n = 12). Qualitative data were analysed using Braun and Clarke’s six-phase thematic analysis, applying a deductive framework informed by the 4Fs. Quantitative survey data were integrated to examine the distribution of stress responses across reported incidents.

**Results:**

Across all data sources, 123 ED staff described 145 coded instances of the 4F stress responses: fight (n=94); flight (n=24), freeze (n=15), and fawn (n=12). Four overarching themes that underpinned these responses were identified: 1) Moral and emotional impact on clinicians, 2) Safety systems interface, 3) Organisational constraints and environment and 4) Patient Complexity. Participants described sustained hyperarousal, anticipatory vigilance, moral distress, emotional withdrawal, and appeasement behaviours arising from repeated exposure to violence. While Code Black responses were viewed as a structured and effective “organised fight” mechanism, this was compromised by inconsistent training, role ambiguity, and limited post-incident support which contributed to burnout, disengagement, and workforce attrition.

**Conclusion:**

Emergency department clinicians operate in persistently threatening environments that drive recurrent activation of fight, flight, freeze, and fawn responses with cumulative individual and system-level consequences. Behavioural emergencies warrant the same level of preparation, standardisation, and organisational support as other high-risk clinical events, such as a Code Blue – a life-threatening medical event with a responding Medical Emergency Team. Trauma-informed education team-based training, and embedded post-incident support are critical to safeguarding staff wellbeing and sustaining the ED workforce.

## 1 Introduction

Workplace violence and aggression (WPV) directed towards healthcare workers is increasing globally and is of significant concern for health systems. Up to 62% of healthcare workers report experiencing some form of WPV, most commonly verbal abuse (58%), threats (33%), and sexual harassment (12.4%) (1, 2). Consistent with findings from Australia, international studies report an escalation in violence and aggression since the COVID-19 pandemic (3–5). Staff in EDs and in psychiatric settings experience higher rates of violence and aggression than in any other clinical setting (6). Nine in ten ED physicians in Australia report weekly exposure to at least one violent incident, with approximately one in two reporting daily exposure (3). Similarly, an Australian survey in 2010 showed that almost 9 in 10 emergency nurses (87%) experienced violence within six months (7); by 2025, the NSW Nursing and Midwifery Association, the state nurses’ union stated this had risen to nearly all nurses (97%) (8). These figures underscore the growing prevalence of workplace violence in EDs. The self-reported psychological and behavioural responses in clinicians to the threat of violence and aggression remain underexplored. Trauma theory conceptualises stress responses as fight, flight, freeze, and fawn (4Fs); these responses manifest in ED staff during and after violent incidents yet have received limited empirical attention.

### 1.1 Background

In EDs WPV emerges from a convergence of system-level and patient-related drivers. System pressures such as overcrowding, prolonged waiting times, and limited access to inpatient beds are frequently cited (9, 10). Patient- and carer-related factors include acute pain, unmet needs (hunger, toileting, liberty) substance intoxication or withdrawal, mental illness, cognitive impairment (including dementia), acquired brain injury (3, 11–15) and acute medical conditions (delirium, sepsis). Family members experiencing distress or fear for the wellbeing of their loved ones have also been identified as performing acts of violence (12, 15–17). Several studies reveal that female staff members and older clinicians are disproportionately affected by violent incidents (4, 14, 18). Additional organisational risk factors include chronic understaffing (4, 19), unrestricted public access within ED environments (15, 19), persistent underreporting of incidents (3, 12, 20) environmental mismatch (21) and poor or ineffective communication (22).

Globally, the COVID-19 pandemic intensified these pressures, coinciding with increased rates of WPV (23), rising mental health–related ED presentations (24), and declining staff morale. According to a Uni Global Union advocacy report, these factors contributed to widespread workforce attrition, resulting in staffing shortages and increased reliance on locum staff and part-time visiting medical officers rather than permanent, fulltime staff (25, 26). Within hospitals, access block, where patients cannot be transferred to inpatient beds due to capacity constraints, disrupt patient flow and prolong ED stays (27–30).

In Australia, reduced access to bulk billing in general practice (when the patient pays nothing and Medicare is billed directly for full payment by the doctor) has driven more people – including aged care residents and patients experiencing mental health crises, to seek care in EDs (31). Consequently, Australia is now seeing >9 million ED presentations annually, with a clear upward trajectory (32). ED attendances in New South Wales (NSW) have increased by 19.2% over the past decade, exceeding population growth (32).

Mental health–related presentations place particular pressure on EDs: in NSW there were 130,151 adult mental health–related presentations in 2024–2025, including an estimated 61,579 involving self-harm or suicidal ideation (33). Patients detained under the Mental Health Act 2007 often experience prolonged ED stays while awaiting specialist assessment. Overstimulating environments, combined with limited communication about expected wait times, can exacerbate distress and the likelihood of aggression, often resulting in a Code Black, organised emergency response to violence, aggression or imminent danger involving restraint and chemical sedation (34, 35). These responses focus on containment and security. NSW Health data further demonstrate prolonged ED bed occupancy by mental health patients, with repeated use of restraint and sedation associated with documented psychological, physical, and emotional harm to both patients and staff (36, 37).

These pressures reflect a broader stress in mental health care provision in NSW. Decades of underinvestment have resulted in prolonged ED waits, limited specialist availability, and critically low numbers of mental health inpatient beds (38, 39). In 2025, the resignation of approximately 500 public sector psychiatrists further destabilised the system, leaving EDs to manage patients with limited pathways for onward care (40). While many have returned as Visiting Medical Officers (time-limited contract workers) or as part-time public sector salaried psychiatrists, there remains a shortage in EDs across the state of NSW. The Royal Australian and New Zealand College of Psychiatrists, emphasises that EDs were never intended as the primary entry point for mental health care; however, under-resourced community services have redirected patients into hyper-stimulus EDs not designed to safely manage behavioural emergencies, increasing risk for both patients and staff (40–42).

Despite the scale of WPV, relatively few studies have centred on the subjective experiences and behavioural consequences in ED clinicians and the toll of exposure to repeated violence and aggression (43). The stress response commonly referred to as fight or flight (44–46) has, over time, expanded to include freeze and fawn (4Fs) (47). The 4Fs can be situated within broader neurobiological models of defensive responding, including the defence cascade, which Kozlowska et al. (48) describe as comprising “arousal, freezing, flight or fight, tonic immobility, collapsed immobility, and quiescent immobility” (p. 264), responses originally observed in animals exposed to threat (48). The autonomic activation and behavioural adaptations that support immediate survival; however, repeated activation has been linked to burn out, chronic stress, cardiovascular and immune dysregulation, chronic pain, anxiety, and depression and substance abuse (47, 49, 50).

Within EDs, clinicians frequently encounter conditions that trigger the 4Fs, yet the manifestations of these responses in staff, during and after aggressive incidents, remain under described.

The 4Fs can be described as follows:

The fight response involves assertively reacting to a threat. It mobilises an action orientated response or hypervigilance to the perceived danger.

The flight response occurs when an individual attempts to avoid or escape the threat by physically leaving the environment or engaging in avoidance, to distract or withdraw from a distressing situation.

The freeze response occurs when the individual believes escape or resistance is impossible. It is typified by physical immobility, reduced responsiveness, or a sense of detachment from one’s body. Individuals may appear disconnected or briefly unable to speak following exposure to threat (51).

The fawn response refers to a trauma-related survival strategy in which an individual attempts to pacify or comply with a perceived aggressor to decrease the possibility of harm (47).

While fight, flight and freeze responses are well established within neurobiological and behavioural research, the concept of fawn is derived primarily from clinical and trauma-informed literature and currently has a less developed empirical evidence base.

### 1.2 Study setting and participant sampling

In 2019, rising concerns about WPV within a local health district in metropolitan Sydney, Australia. prompted a program of research across three hospitals and four ED sites. Early qualitative work on staff experiences during Code Black incidents (52) led to a series of safety and care interventions in 2020. This included the introduction of the Behaviours of Concern (BOC) chart to heighten objective awareness of agitation and imminent aggression (53, 54); de-escalation skills; standardised equipment contained in the black box, including sedation medication and restraints, and clearly defined roles during restraint and sedation.

In 2022, a series of focus groups was conducted to further explore staff wellbeing (55). Finally, in 2023 a post intervention study was undertaken to find out how the Code Black program of change implementation was faring (56) (56). While early findings indicated initial success, subsequent NSW Bureau of Health Information data from 2025 demonstrated an increase in both the frequency and duration of restraint episodes (36).

This paper presents a secondary analysis of data from three research phases: 2019 semi-structured interviews on staff experiences of violence and aggression (52); focus group data on ED staff mental health, wellbeing, resilience and culture (55); and a study of barriers and facilitators to sustainable implementation of a restructured Code Black response, including associated processes and training (56). Given escalating ED violence internationally, we reanalysed these datasets using the 4Fs framework to identify cross-cutting themes not evident in isolation. This synthesis serves the same purpose: deepening understanding of how violence affects ED clinicians and identifying training improvements within the ED’s environmental, systemic and cultural constraints, ultimately supporting staff and patient safety and wellbeing. While some qualitative comments and quantitative results are reproduced, the 4Fs framework offers a new interpretive lens, adding knowledge to this area.

### 1.3 Inclusion and exclusion criteria

Inclusion: Clinical, public-facing and security staff involved in managing aggressive patients.

Exclusion: ED staff not involved in managing aggressive patients.

### 1.4 Recruitment

Staff were recruited for interviews and focus groups via manager emails. Interviews were conducted in person (in a room separate from the ED) or via Teams, with participants choosing their own space. The survey was distributed via managers or accessed through flyers in ED staff common areas. Survey invitations were not distributed to a defined list of individual staff members, and so did not generate a record of the number of eligible staff who received or viewed the invitation. The total number invited therefore could not be determined, and a survey response rate could not be calculated.

In this mixed-methods study we review data from a survey, interviews and focus groups spanning 2019 – 2023. We address four questions:

### 1.5 Research questions

- Are fight, flight, freeze, and fawn responses evident among emergency department (ED) staff when managing potentially aggressive or violent patients?
- What are the drivers of the 4Fs?
- How are the four ‘F’ (fight, flight, freeze, fawn) responses manifested by ED clinicians in real-world clinical practice during behavioural emergencies and what are some of the consequences?
- What strategies can be implemented to support ED staff before, during, and after exposure to behavioural emergencies?

## 2 Method

This study used qualitatively driven, multistage mixed methods secondary analysis design. Qualitative interviews, focus-groups and survey free-text data were prioritised, while quantitative survey data provided descriptive evidence to support and contextualise the qualitative findings. Integration was undertaken in the secondary analysis by aligning findings from the different data sources within the common conceptual framework of fight, flight, freeze and fawn (4Fs).

Specifically, the study drew on four data sources: individual, semi-structured interviews conducted in 2019 by JD and MB (n=20), six focus groups held in 2021 by JD and MM (n=30 participants), a survey administered in 2023 by JD and MM (n=61) and follow-up semi-structured interviews undertaken in 2023 by JD (n=12). Reporting was guided by the Consolidated Criteria for Reporting Qualitative Research (COREQ) for the interviews and focus groups, the Strengthening the Reporting of Observational Studies in Epidemiology (STROBE) checklist for the surveys and Good Reporting of a Mixed Methods Study (GRAMMS) for the mixed methods design and integration (57–59).

The survey was developed by the research team and piloted prior to distribution; formal psychometric validation was not undertaken. The interview guide was also developed specifically for this study; formal validation was not undertaken. Both instruments are provided as Appendices 1 and 2 in (56). Data saturation was reached by interview 18 and confirmed by interview 20 (2019), and after six focus groups (2021). In 2023, the post-intervention survey with qualitative comments and 12 interviews provided adequate insight.

Qualitative data were analysed using Braun and Clarke’s six-phase thematic analysis, starting inductively before applying a deductive framework based on the 4Fs (fight, flight, freeze, fawn) responses (60), once their recurrence emerged. Quantitative survey data were analysed descriptively. The qualitative and quantitative datasets were initially analysed separately before integration.

Participants contributed data concerning behavioural emergencies and workplace violence across multiple phases of programme implementation. The purpose of this secondary analysis was not to evaluate intervention effectiveness or measure change over time, but rather to identify recurring trauma-response patterns across the combined datasets. Accordingly, the analysis focused on commonalities in clinicians’ experiences and behavioural responses to workplace violence irrespective of collection phase.

Findings are therefore presented using a theme-by-theme narrative-weaving approach, whereby descriptive survey results are integrated with qualitative accounts to explore recurring manifestations of fight, flight, freeze, and fawn responses (61). Survey findings are presented alongside corresponding qualitative themes and through a joint display (**Table 1**). Qualitative data remained the primary source of interpretation, while quantitative findings were used to support, contextualise, and qualify the qualitative analysis.

**Table 1:** Joint display of coded qualitative themes and codes, pooled across INT2019, FG2021, SUR2023 and INT2023, with corresponding SUR2023 survey results presented alongside where available.^^1^^.

Themes	Categories	Codes	Survey data 2023
Moral and emotional impact on clinicians *Fight (n=39)*	*Internal reactions*	•Compelled to act (n=22) •Internal struggle (n=11) •Unsupported (n=4) •Oscillating Fight-Flight responses (=2) ^^2^^	91.8% of ED staff cared for a patient experiencing behavioural disturbance in the past 3 months. 78.7% had been assaulted or threatened at work Staff felt challenged: Clinically – 57% Emotionally – 41% Ethically – 28.5% Did not feel challenged – 28.5% Post- Code Black effect on wellbeing: Does not affect my wellbeing at all – 39.5% Has a negative effect at the time – 54% Has a negative effect after – 23%
*Flight (n=18)*	*Moral injury*	•Internal emotional struggle (n=4)	Post- Code Black, staff sought support from: ED colleagues – 64.5% ED Managers – 15.5% Employee Assistance Program – 5% Private psychologist/psychiatrist/counsellor – 3.5% Friends and family – 46% Other – 10%
	*Loss of control*	•Staff turnover (n=8) •Time out (n=3) •Loss of control (n=3) •No support (n=3)	no corresponding survey item
*Freeze (n=15)*	*Shutdown*	•Fear (n=2) •Shutdown/Exhaustion (n=13)	no corresponding survey item
*Fawn (n=3)*	*Self-regulation*	•Anticipating trouble/self-moderation (n=3)	no corresponding survey item
Systems interface *Fight (n= 33)*	*Standardised organisational response*	•Organised fight response to Code Black (n=11)	Level of confidence managing a Code Black: Identifying Behaviours of Concern (87%), Activating Code Black (85%), Identifying when to restrain (88%), When to sedate (82%) How to sedate (85%), Post-sedation care (83%) Knowing roles/responsibilities (79%)
	Non standardised organisational response	•Staff in fight mode agitating patients (n=4) •Role conflict (n=9) •Unsupported means loss of staff (n=2) •Noncompliance with Code Black procedures (n= 7)	Number of staff who did not report the incident: 25% Was the incident debriefed? Yes 19.5% No – 80.5% Limited commitment to using the Code Black tools: Did not believe the BOC chart identified behaviours of concern 38.5% (n=23) Did not find it improved patient management 42.5% (n=26) Did not think it reduced ED violence, 47.5% (n=29) Intention to use the sedation algorithm: mean = 5.34/10 (53.4%), SD = 2.74, median = 6, n = 59. Intention to use the restraint policy: mean = 6.87/10 (68.7%), SD = 2.51, median = 7, n = 60
*Flight (n=6)*	*Non standardised organisational response*	•Unsupported (n=3) •Role conflict (n=3)	no corresponding survey item
Organisational constraints *Fight (n=22)* *Fawn (n=1)*	*Environment*	•Anticipatory arousal/fight readiness (n=16) •Unpredictable environment (n=6) •Unsupported (departmentally) (n=1)	How safe do you feel at work? Always – 11.5% Usually – 65.5% Sometimes 19.5% Never: 3.5% Staff support received after a Code Black: Debrief – 29.2% Counselling – 6.5% Nil – 64.5% Other 4%
Patient complexity *Fawn* (n=8)	*De-escalation*	•Offering reward for less trouble (n=6) •Not acting for less trouble (n=1) •Unsupported (n=1)	no corresponding survey item

1 Counts reflect the number of coded instances, not incidents or participants, and are not mutually exclusive across codes; see Method for further detail.

2 Two instances described extended bouts of fight behaviour that oscillated with strategic withdrawal; both were coded under Fight because the situation was ultimately resolved through sedation and restraint.

The counts presented in **Table 1** represent coded instances rather than incidents or participants. A single account could generate multiple, non-mutually exclusive codes across the four response categories. As the volume of analysable text varied substantially between data sources (for example, a 90-minute interview compared with a brief survey response), these counts indicate the relative prominence of themes rather than prevalence within a defined population.

The research team met regularly between October 2025 and May 2026 to review coding decisions, reconcile discrepancies, and refine the analytic framework. To ensure a shared analytical foundation, the team first defined the 4Fs and applied these definitions consistently across data sources. All qualitative data were distributed among the four authors (JD, MM, MB, GdeM), each of whom independently analysed their assigned material to identify instances reflecting the 4Fs; this work was then cross-checked across the team to support dependability and confirmability. Any disagreements were resolved through discussion among the research team, and a decision log was maintained to document key discussions and the rationale underpinning coding and theme development. Extracted data were organised into source-specific spreadsheets (INT2019, FG2021, SUR2023, INT2023), with participant identifiers (P#) assigned where available.

Examples were then consolidated into four master datasets corresponding to the 4Fs categories. The research team subsequently coded the data, developing a comprehensive coding framework in separate spreadsheets. These codes were grouped into categories and broader themes as they developed. The initial coding results were checked by all four researchers (JD, MB, GdeM, MM) to ensure the accuracy of the interpretation of the data and discuss and refine major and minor themes. Through iterative team discussion, the authors reviewed, refined, and confirmed the final codes categories, before reaching consensus on the overarching themes (See **Figure 1**: Methodology Process).

**Figure 1:**
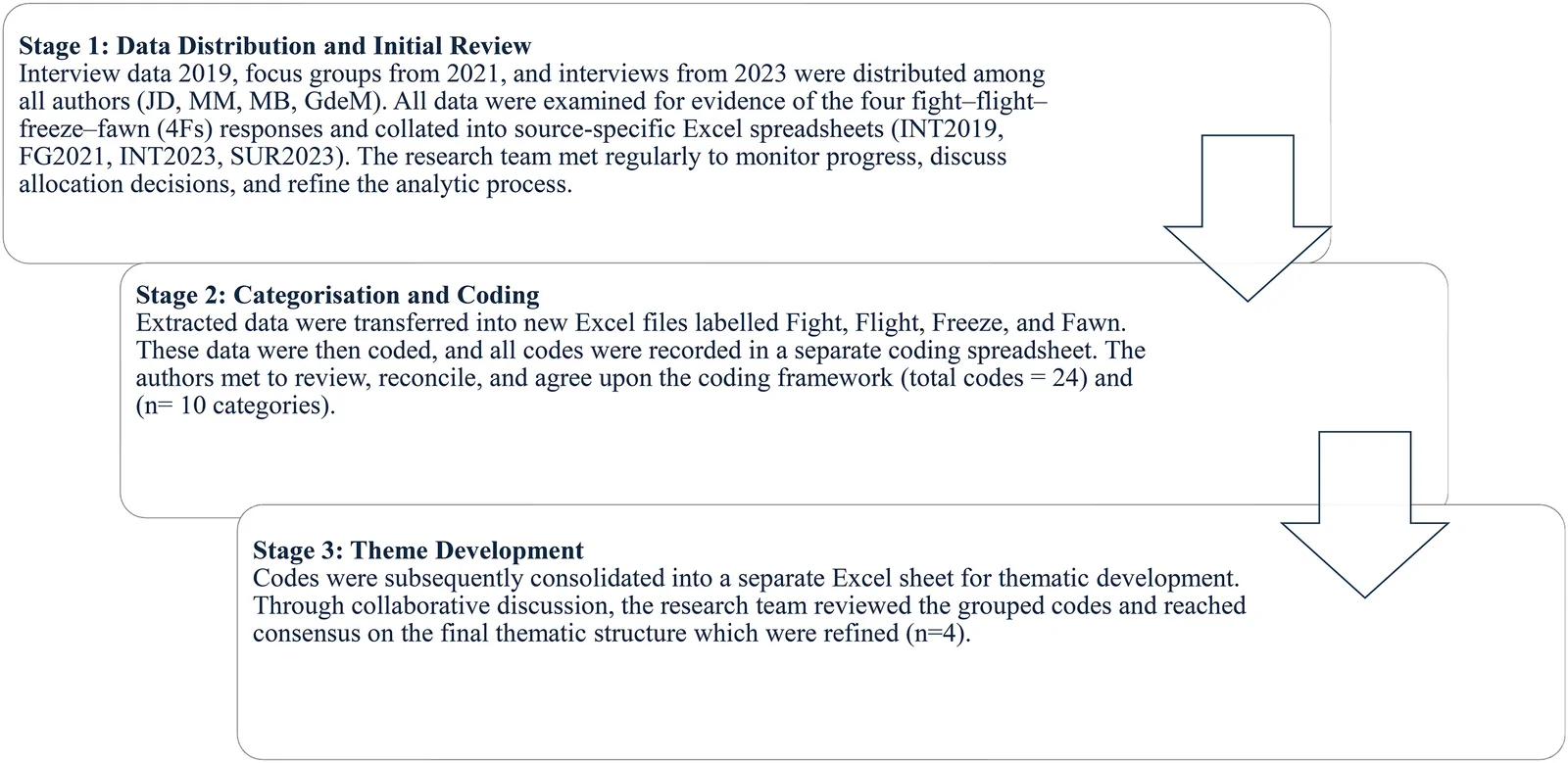
Methodology process.

### 2.1 Ethics

Multi-site ethics approval was obtained from the Human Research Ethics Committee (HREC) at Western Sydney Local Health District Ethics Ref: Application ID: 2019/ETH00598. The original HREC approval was granted on 15 May 2019. The 2021 amendment, approved on 17 May 2021, extended the protocol to cover the 2023 survey and follow-up interviews. On 20 August 2026, the WSLHD HREC chair confirmed that secondary analysis of the pooled 2019–2023 dataset for the present study falls within the scope of this approval and its amendment.

### 2.2 Reflexivity, rigour and trustworthiness

The research team comprised four authors with differing relationships to the clinical setting: MM, ED staff, offered insider contextual and clinical insight, while JD, GdeM and MB, as outsiders, balanced potential insider bias and supported critical distance during analysis. Authors adopted as neutral a stance as possible while remaining attentive to MM’s clinical insights. All interviews were conducted by non-ED research members (JD, MB).

In 2019 interview transcripts were returned to participants for comment (52). COVID-19 and staff shortages affected recruitment during 2023, but sufficient data, supported by survey responses, provided adequate insight. Member checking occurred informally, with interviewers summarising and confirming participants’ comments during data collection (56). The coding process conducted by the four authors to ensure rigour, can be seen in Methods. Staff quotes are used extensively throughout the findings to preserve participant voice and support credibility and trustworthiness, supplemented by relevant survey data.

## 3 Findings

Across three phases (interviews 2019, focus groups 2021, survey and post-intervention interviews 2023), 123 ED staff contributed data concerning behavioural emergencies and workplace violence. Findings are presented using a theme-by-theme narrative-weaving approach in which descriptive survey results are integrated with qualitative accounts. This program of research revealed that their responses to behavioural emergencies in the emergency department were multifaceted, dynamic, and shaped by both individual and organisational factors. Participants represented nursing, medical, administration and security, and spanned a wide range of emergency experience. The participants were proportionally representative of groups working within the ED. Gender was self-reported by survey participants, using the categories Male, Female, and Rather not say. Sex was not separately assessed. (See **Table 2**, Participant Demographics).

**Table 2:** Participant demographics.

Characteristics *n* (%)	Qualitative phase n (%) interviews 2019 total n=20	Qualitative phase 6 focus groups 2021 total n=30	Quantitative/qualitative phase *n* (%) (survey) 2023 total n=61	Qualitative phase *n* (%) interviews 2023 total n=12
Site	Site 1 (n=5), Site 2a (n=5), Site 2b (n=5), Site 3 (n=5)	Staff from all sites attending Site 1		Site 1 (n=5), Site 2 a & b (n=3), Site 3 (n=4)
Gender				
Male	n=10 (50)	n=7 (23.3)	n=23 (37.5)	n=7 (58.3)
Female	n=10 (50)	n=23 (77.7)	n=35 (57.5)	n=5 (41.7)
Rather not say			n=3 (5)	n=0 (0.0)
Profession				
Administration officer	n=1(5)	n=0 (0.0)	n=2 (3.3)	n=0 (0.0)
Physician	n=6 (30)	n=4	n=21 (34.4)	n=4 (33.3)
Nurse	n=13 (65)	n=26	n=34 (55.7)	n=6 (50)
Security	n=0 (0.0)	n=0 (0.0)	n=3 (5)	n=2 (16.7)
Allied Health	n=0 (0.0)	n=0 (0.0)	n=0 (0.0)	n=0 (0.0)
Other	n=0 (0.0)	n=0 (0.0)	n=1 (1.6)	n=0 (0.0)
Experience working in the ED (years) *n* (%)				
1-5	n=13 (65)	The medical staff had a median 17.5 years’ emergency experience compared to 7.5 years for nurses.	n=17 (28)	n=2 (16.7)
6-10	n=6 (30)		n=12 (19.5)	n=6 (50.0)
11-20	n=1 (5)		n=22 (36)	n=3 (25.0)
21-30			n=9 (15)	n=0 (0.0)
>30			n=1 (1.5)	n=1 (8.3)

Using the trauma-informed framework of fight, flight, freeze, and fawn (4Fs) as an analytic lens, responses were identified across all four domains, with varying frequencies and patterns of manifestation. Thematic analysis generated 24 codes, 10 categories, and 4 themes. Because these instance counts are shaped by the volume of codeable text each data source generated as well as by the frequency of the underlying practice, they are presented below as an indication of relative prominence within the dataset rather than as proportions of a defined population (see Method). The four themes that captured how trauma responses were experienced, enacted, driven and sustained by clinicians within emergency care settings were: 1) Moral and emotional impact on clinicians, 2) Safety systems interface, 3) Organisational constraints and environment and 4) Patient Complexity (physical, psychological, socio-economic factors, culture, language, education). When the episodes of threatening behaviour were analysed all four of these themes come into play to varying degrees (See **Table 1** Thematic analysis).

### 3.1 Moral and emotional impact on staff

Across all four stress responses, staff described a significant moral and emotional burden associated with managing behavioural emergencies. In 2023, 91.8% (n=55) of ED staff responding to the survey had cared for a patient with behavioural disturbance in the previous 3 months, and 78.7% (n=48) had been assaulted or threatened at work. Staff felt challenged clinically (57%, n=35), emotionally (41%, n=25) and ethically (28.5%, n=17). While 39.5% (n=24) reported no effect on wellbeing, more felt affected at the time (54%, n=33) or afterward (23%, n=14). Qualitative data below provide further insight.

Within fight responses (n=39) staff frequently reported feeling compelled to act, often accompanied by internal conflict and perceptions of being unsupported. They expressed tension between professional duty and personal safety. Feeling compelled to act was described when staff discussed initiating Code Black to protect the safety of patients, colleagues, the broader emergency department environment and themselves. Staff explained this in the following quotes:

It takes a lot of energy and it’s emotional I think for a lot of staff because you’re holding people down and restraining them and it’s for their own benefit so they’re not harming them or us really. (P6, nursing staff, INT2019)

…it was a split second, where you had to change your frame of mind from, you know, fight or flight to I need to do my job… (P20, nursing staff INT2019)

So, I have to take action … we end up having to chemically sedate them or we can’t do anything… (P5, nursing staff INT2019)

A participant vividly conveyed the chaotic and intense emotional nature of their “fight” response when they said:

‘I’m running around like a psycho just trying to survive. (P18, nurse educator INT2019)

The following quotes describe teams working at cross-purposes, with both clinical and security staff feeling entirely unsupported. This caused tension and internal conflict when staff perceived a real threat to their personal safety:

… Afterwards when I realized security weren’t going to help me; I had to take things into my own hands … I just managed to tip him off balance and everyone fell on top of him. As far as injuries went, I had an almost dislocated jaw … a few cuts inside of the cheek from where he punched me … I actually had to go see a dentist and had a tooth removed. Yeah, so I couldn’t eat properly for about two weeks (P8, nursing staff, INT2019)

‘From what I’ve experienced like, not all the (clinical) staff are willing to respond … coming from a security perspective, I feel like … all the responsibilities thrown onto the security officers’ shoulders to respond and lead the response. I don’t feel there is, like, a team effort. (P14, security staff, INT2023)

Flight responses (n=18) were frequently linked to moral injury with staff describing withdrawal behaviours such as taking time out or leaving the workplace, reflecting loss of control and ongoing distress. Staff sometimes physically removed themselves during Code Black due to fear; or afterwards to recover and regain composure before returning to work. Here are some reactions described by staff to incidents that occurred indicating fear, anxiety, denial and withdrawal:

I remember everyone being heightened, for the whole week … it was a lot of staff members who went into their shell and started getting that the whole PTSD feel right? (P17, nursing staff, INT2019)

There was definitely some apprehension with going back into that environment, knowing what had happened. (P20, nursing staff, INT 2019)

God … I, I’ve got anxiety, I think … But I’ve had a couple … that’s made me scared or, you know, apprehensive … It’s just my nature and I find it easy to just keep things rolling over. And you know, try to stuff it to the back of your head and not think about it…. you’re very heightened, so … It would be near impossible to turn around and be, you know, the same person you were … you know something extreme happened … someone got hurt … You know, that would probably stay with you. (P3, medical staff, INT 2023)

Flight responses were observed both in the immediate aftermath of behavioural emergencies and as longer-term patterns of disengagement. Although some clinicians appeared to cope with emotional distress in the short term, these responses often evolved into withdrawal behaviours over time. A persistent sense of loss of control was frequently reported and was perceived by clinicians to contribute to workforce attrition:

losing control … over our environment and our work practices … you can’t provide sometimes basic care, because of factors out of your control. And that’s the biggest threat. That’s why people leave. That’s why a lot of people burn out because of the you know, substandard care (P2, medical staff, FG62021)

Interview data pointed to significant post-COVID workforce attrition, which intensified already chronic understaffing and workload pressures. These conditions were linked to increased reports of illness and avoidance behaviours, reflecting a broader sentiment among clinicians that workplace conditions were unlikely to improve:

Then somebody will be sick and then the rest are carrying it and then that group is then kind of feeling even more burnt out, somewhat resentful. And the next thing you know (there’s) this cycle of work avoidance… (P.4, medical staff, FG62021)

I’m driving so angry, because I’m just so disappointed, so overwhelmed, just cranky with the system. That I go home … I’m going to apply for other jobs. I can’t do this anymore. (P1, nursing staff, FG52021)

You’re just so burnt out. And like, you’re just like, what’s the point? Like, they’re not going to change anything anyway … Yeah. I think we always have that view and I had it shit today; it’s going to be shit tomorrow. Yep, nothing’s going to change… (unidentified nursing staff, FG32021)

We go through so many shifts where it’s just like, you’re overworked, overworked, overworked, no one cares … they’re not providing us anything to help with that, like staff, or even trying to create space for us in the ward when, when we have bed blocked and trying to rectify that… (P4, nursing staff FG32021)

Freeze responses (n=15) were characterised by psychological shutdown, including fear, burnout or internal struggle, suggesting cumulative effects of repeated exposure. Freeze responses were most evident when ED staff felt overwhelmed by fear or a profound sense of helplessness during Code Black events. While freezing could occur in response to immediate threat, as manifest by a physical shutdown or an intrapsychic closing down of processing the situation, the interviews revealed it more commonly presenting in the cumulative erosion of personal and professional identity. Typically, this was associated with burnout. Staff described feeling mentally exhausted, anxious, apprehensive, and emotionally depleted, consistent with compassion fatigue and nervous system shutdown. This chronic freeze response was marked by a reduced capacity to engage or act, extending beyond the workplace to their personal lives. The inability to act in the immediate moment of threat is illustrated in the following harrowing description of a drug-affected young man threatening staff and other patients; notably the account highlights the vulnerability experienced by female staff:

*And probably over the period of about an hour and a half, he was becoming agitated, you know, wriggling around the bed, didn’t want to lie flat, you know, carrying on, are these parents probably getting a bit overly aggressive with me … And, you know, swearing, … all I can say is … I’m here to keep you safe. Really sorry. I’m waiting for doctor to come … I very regularly went to the doctor and said, ‘Look, I’ve got this gentleman … He’s escalating. Can you please come and see him and explain to him what’s going on? … I’m worried that something’s going to happen’ … it was a very chaotic, busy environment … it was an afternoon shift, so, it’s always a bit feral. And there’s lots going on … eventually, he started to get really agitated and sort of thrashing around in his bed and yelling and swearing and the security came and he was in a bed space next to another little old man and his wife … his parents were there trying to calm him down … the gentleman got up on the bed standing and was threatening us, he had an IV pole … [the patient attacks the security staff] … me and this other female staff member just stood there frozen. And I thought, if these two six-foot something men, sort of, you know, are getting pummeled, what am I gonna do? … And then all I could remember hearing on the overhead is like, can we get any male staff member to beds 21 and 22*. (P18, nursing staff, INT2019)

Longer-term freeze responses were most expressed as emotional detachment and disengagement. Clinicians described a process of “shutting down” with spillover effects that, at times, extended into their personal lives:

Sometimes there’s a point where you just reach complete deflation. You just come back, and we keep doing this thing over and over again, and you go home and you’re tired, and you’re frustrated, and you’re sad. And you realize, like, okay, I’m not even looking after myself or caring for myself, and I’m not my normal self anymore. (P2, nursing staff, FG52021)

We debrief, try not to take it home, but the reality is, we do … like my husband thinks and says, ‘I don’t understand how you can be an emergency physician, you can’t make a decision’. But that’s because I come home and I’m like I’m done making decisions. (P3, medical staff, FG62021)

These accounts show that freeze can affect clinical decision making. P18’s “we just stood there frozen” reflects a delay of risk assessment during the acute phase exactly when help-seeking was needed — indicating freeze can suspend intervention during emergencies. The chronic shutdown P2 describes can plausibly extend to reduced situational awareness and engagement and is consistent with the literature on emotional shutdown and compassion fatigue (48, 62). Freeze functions therefore as both an acute safety risk and a chronic driver of disengagement.

Fawn responses (n=3) involved forms of self-moderation, whereby clinicians consciously altered their behaviour to reduce the risk of escalating aggression, often at personal or professional cost. These responses functioned as behavioural strategies to manage potentially volatile situations and included pacifying, complying, or distractive approaches. Careful self-moderation was described as a deliberate and ongoing process in which clinicians regulated both verbal and non-verbal behaviours to avoid exacerbating distress in patients displaying signs of agitation or tension. This process sometimes involved temporarily disengaging from interactions, such as stepping away from the patient, allowing clinicians to regulate their own emotional responses while mentally recalibrating and developing more effective strategies to support patient cooperation and de-escalation.

The following accounts illustrate how nurses adapted their behaviour through fawning and self-moderation as a strategy to avoid triggering patient anger:

To me as a triage nurse, you have to be sort of sensitive with what you say. You don’t want to push them over because sometimes one little word that you say, they can get angry at you. (P4, nursing staff, INT2019)

You’ve just got to kind of take it, and you know, you can feel yourself getting - because we’re human. We yeah, if someone’s abusing us you want to kind of … you can’t - you’re there to look out for their best interests. You (have) just got to take a deep breath and just ‘okay, it’s okay.’ You know, let’s try to figure this out. Let’s try a new strategy. Let’s go for a walk. (P6, nursing staff, INT2019)

### 3.2 Safety systems interface

Staff responses were strongly linked to how ED interacted with the safety systems set up within the organisation and shaped clinician responses across two of the four trauma response patterns—fight and flight.

Fight responses (n=33) reflected both structured, standardised team-based interventions and less consistent, non-standardised approaches to managing aggression. Standardised team-based interventions were predominantly the activation of an organised response to aggression, called Code Black. Non-standardised responses included staff behaviours that inadvertently escalated situations, role conflict, and non-compliance with protocols. Perceived lack of support also contributed to workforce strain, including loss of staff.

In situations where violence persisted despite de-escalation attempts, staff frequently escalated their response by calling a Code Black and applying physical restraint. This formalised, systematised “fight” response was perceived as effective due to the structure it imposed including the application of the BOC chart; de-escalation skills and the use of standardised equipment, medication and restraints contained in the black box; and clearly defined roles during restraint and sedation. This structured approach supported controlled safe restraint, as described by a nurse:

The BOC score … helps junior staff … medications are all in the box … we do really well at restraining patients … in a controlled way. (P1, nursing staff INT2023)

Generally, survey respondents selected confident to very confident in applying the process of this standardised approach measured across Code Black related tasks (n=61): identifying BOC (87% n=53), activating Code Black (85% n=52), identifying when to restrain (88% n=54), when to sedate (82% n=50) and how to sedate (85% n=52), post-sedation care (83% n=50), and knowing roles/responsibilities (79% n=48).

While Code Black protocols provide a framework for managing workplace violence, their enactment was often complex and iterative requiring repeated cycles of withdrawal and reengagement to restore safety. This fight response progressively depleted staff’s physical, cognitive, and emotional resources as described in this prolonged Code Black incident:

… I guess it just escalated to a point that we couldn’t control. Everyone was backing back for their own safety … And then, he kept yelling at the doctor who was obviously pretty upset, who had to then be removed from the room … then at that point he stood up and produced a shiv (homemade knife)….at that point everyone retreated from the room … while that was happening one of the doctors actually sat in his chair in the middle of the room and everyone else cleared out and he was trying to verbally deescalate him … few of us were sort of on the side pushing equipment out, as best as we could, to try and clear the room. (P.16, nursing staff, INT 2019)

Noncompliance with Code Black protocols was reported and attributed to multiple factors, including high workload, lack of training, and poor coordination. In some cases, senior clinicians deliberately deviated from established procedures to manage incidents autonomously, while newer staff lacked familiarity with the BOC chart, limiting appropriate responses. ED staff showed limited commitment to the BOC chart: 38.5% (n=23) did not believe it identified behaviours of concern, 42.5% (n=26) did not find it improved patient management, and 47.5% (n=29) did not think it reduced ED violence. Intention to use the sedation algorithm was moderate (mean 5.34, SD 2.74, median 6, n=59), while intention to use the restraint policy was higher and more consistent (mean 6.87, SD 2.51, median 7, n=60), skewed toward the upper end of the scale.

Poorly coordinated team-based practice further contributed to confusion, with some staff attending Code Black activations as observers rather than active participants, inadvertently increasing disruption. These instances, described by two medical staff, shed light on issues of non-compliance which cause inconsistency and confusion:

I kind of know what to look for. But I don’t know the chart that you’re referring to. (BOC chart) (P8, medical staff, INT2023)

Once one of them said “nup, not in my department, it’s just too busy”. It’d be far quicker to just sedate them, which I felt was not in, you know, this is against the policy, but I could understand where they’re coming from (P8, medical staff, INT2023)

Yeah! You get all the juniors, you get all the registrar’s, you’ll get the senior nurses because that, like the NUM and the team leader will turn up (at the sound of a Code Black alarm) (P3, medical staff, INT2023)

And I think it’s very dependent on the boss. (if Code Black protocols are applied) (P3, medical staff, INT2023)

Post-incident, at least 25% (n=15) of staff did not report the incident, and 80.5% (n=49) were not debriefed. Staff sought support from ED colleagues (64.5%, n=39), ED managers (15.5%, n=9), the Employee Assistance Program (5%, n=3), a private psychologist/psychiatrist/counsellor (3.5%, n=2), or friends and family (46%, n=28).

I'll say that the clinicians are looking for a fight, but like, a bit of aggression or asserting their power…who are either trying to assert their power don't have the skills …it's like the minority, but that minority, if they're on a shift together, will agitate (P18, nursing staff, INT2019).

Flight responses (n=6) further reflected system-level challenges, with staff reporting disengagement as coordination of Code Black did not always go as planned. Systemic failures, such as role confusion within the Code Black team and inconsistent training across clinical and security staff left staff feeling unsupported during an aggressive incident. Limited induction meant that casual security staff were not familiar with Code Black processes. In some cases, security personnel failed to intervene as expected, while conversely reporting that responsibility was disproportionately shifted onto them without adequate patient or clinical context. Uncoordinated intervention increased risk during Code Black events, contributing to staff perceptions of exposure and vulnerability with inconsistent compliance and coordination undermining perceived support during critical incidents.

The issue of untrained security staff managing Code Black was frequently mentioned by clinical staff as these staff were often unfamiliar with local Code Black protocols:

We need these (security) staff trained in de-escalation rather than coming in too late or coming on too hot and heavy and making the situation worse for the pt, staff and other pts. (nursing staff, SUR2023)

Quite unsafe, because their security guards didn’t know what to do and had to take a lot of direction from us. The (supervisor) who was watching was quite appalled and had to show them where restraints were … how to put restraints on … how to use the Stryker bed and where to put restraints on. (P8, medical staff, INT2023)

### 3.3 Organisational constraints and the environment

Organisational constraints and environmental conditions shaped clinician responses across two of the four trauma response patterns—fight and fawn. The heavy cognitive load, unpredictability and sustained operational pressures of overcrowding, prolonged waits, and limited access to mental health professionals created a persistently high-arousal environment, priming staff for a fight response to perceived or actual threats. Here a nurse describes the overwhelming nature of working in the ED environment:

…and if you’re got so busy, got caught, caught with a lot of stuff that you need to do. And then they call, “how come you didn’t bring this patient yet? How come this patient? How come you didn’t? How come she’s not admitted yet? Like, how come this wasn’t done? So standard? And how can that occur? How come a covid swab wasn’t done for the parents? How come the results are not back?” (FG4, nursing staff, 2021)

Fight responses (n=22) were evident in how staff prepared for potential aggression. They described actively scanning their environment, anticipating escalation and mentally rehearsing defensive actions (identifying exits or potential means of protection in their environment). For this doctor, exposure to repeated threats had shifted to diffuse anxiety, marked by ongoing anticipation of potential environmental danger in everyday practice:

You know, when things do get overcrowded, access block, aggressive patients you go to work being anxious. Is something bad going to happen today? (FG6, medical staff, 2021)

Being in a constant state of alert due to the unpredictable nature of the ED and primed for any rapid escalation into aggression, this nurse demonstrates how hypervigilance is enacted through continuous environmental risk assessment and the mental rehearsal of defensive actions:

I always enter an area and think, where’s my exit? What bit of equipment could I hit someone with, if I’m threatened … I have that thought, to my mind all the time…., the degree of unknown is so high, that in any at any point in time, something could escalate to, you know, 100 in seconds, especially when you’re working in, you know, the triage areas. (P18, nursing staff INT2019)

Participants’ perceptions of whether it was a safe work environment were mixed. Survey respondents reported that 31% (n=19) did not agree that the ED promoted a culture of safety and security. The majority 77% (n=47) reported always or usually feeling safe at work, 23% (n=14) indicated they sometimes or never felt safe. Staff explain how organisational constraints make them feel unsafe and unsupported by organisational leadership in the following quote:

They’ve (managers) got their own pressures that we can understand. But I don’t think they’ve seen the numbers and what we’re seeing, but they’re not actually physically seeing what we’re dealing with, like, for example, like five mental health patients in our ambulance offload, two of them escalated. They’re not seeing that, they just see the patient’s name, what they come in with. That’s it. They don’t physically see what we have to deal with. And I think they just think, oh, okay, ED, they’ve only got 40 patients in the waiting room. Yep, that’s okay. But they don’t see there’s only one nurse to those 40 patients, and they are quite very unwell patients. And I think they need to come down and physically, instead of sort of giving us pizzas and here you go! (FG3, nursing staff, 2021)

Fawn response (n=1): At a systems’ level the ED operates as the primary interface between the public in acute distress and the broader hospital shaped by pressures across the whole organisation. Within this context, staff described engaging in departmental regulation to manage persistent frustration, delays, and competing demands. This frequently manifested as fawning behaviours to stabilise the larger system in which ED functioned. Such behaviour was characterised by appeasement, placation, and conflict management strategies. Clinicians often recognised their role as one component within a broader, often fragmented healthcare system, requiring continual adaptation to conflicting priorities. These behaviours were adopted to manage risk and preserve patient flow within the hospital system. Despite dissatisfaction with the system, one doctor described the balance required to maintain cooperation and to avoid ‘blowback’:

There’s this general level of dissatisfaction with the system, and we’re the face of the system … think also like we’ve talked about this in terms of the planning days but about redefining ourselves … because we spread ourselves so finely … but to redefine ourselves as a specialty and the core business that I think would help people. Because … that gives you back a sense of control that you practise, which is like a big key, but again very difficult to do because we have to work in the whole system, so we can’t unilaterally say we will do this, we won’t do this. And no negotiation. We can’t hold the system to ransom like that because they’ll just punish us in other ways. (P2, medical staff, FG6 2021)

### 3.4 Patient complexity

Patient complexities –including comorbidities, psychosocial drivers, substance use, cultural factors, and communication challenges – are multiple influencing factors that might affect patient behaviour and that critically shaped clinician responses across one of the four trauma response patterns—fawn. Fawning is a behavioural strategy used to moderate a potentially aggressive situation that might involve pacifying, complying, or distracting behaviour. These behaviours can be seen both in relation to individuals who are threatening, or indeed at an organisational level to maintain security and stability.

Fawn responses (n=8): Fawn strategies used included prioritising the patient to be seen sooner and offering informal ‘privileges’ in exchange for cooperation. While such actions may function as effective de-escalation techniques, some staff regarded them as inadvertently reinforcing problematic or aggressive behaviour. The various types of fawning behaviour are reported here by nursing staff:

you’ll find a lot of mental health patients would love to go out for a cigarette a lot … it’s usually generally safer with a security guard to be out there and watching him. So, we get security come out just have a smoke outside and they’re just glad that from that situation and calmed down a bit and come back in … we end up, what’s the word…? Bargaining? if you will, yeah, but if you behave … we’re happy for you to go out with cigarettes and security guards, help you keep calm and stuff that, but you need to help us out too. If you obviously are aggressive and this is dark, and we’re not going to reward bad behaviour. (P17, nursing staff, INT2019)

Or the more exasperated response that somehow such patients might jump the queue:

They normally get rewarded by being seen, because they’ve now caused a scene. (P5, nursing staff, INT2023)

Or in this case asking a patient to leave the ED is seen as a reward for aggressive behaviour rather than the patient having to accept responsibility and the consequences of his aggression:

The first incident I spoke about, I was quite upset about that for a long time, and it wasn’t necessarily the fact that it had happened. It was more the follow-through … only myself and one of my other colleagues were present at the time because it was early in the morning and everyone was getting medications and everything. But, after we pressed the button, they came running they asked what happened I gave him like, a regurgitation of what happened too quickly. I think I was pretty visibly distressed by it and all that happened was that they took the restraint off and said “Leave” it wasn’t, you know, “You just tried to assault one of my nurses, seriously. We’re going to call the police”. It was just “Leave” and then … he went on to break our equipment … I just feel like sometimes patients get away with it. And so, they think that it’s acceptable behaviour. (P16, nursing staff, INT2019)

## 4 Discussion

Violence and aggression in EDs are of rising concern for healthcare systems worldwide. ED systems are dogged by long wait times, limited access to beds which block patient flow and staff shortages (3, 9, 10). In Australia, these challenges unfold within an environment of sustained operational pressure, compounded by shortages in community mental health services, psychiatric availability and inpatient mental health beds. These system level tensions amplify patient distress and increase the likelihood of behavioural escalation and WPV. (41).

This study identifies that under these circumstances, ED staff experience and exhibit behaviours associated with *fight, flight, freeze, and fawn (4Fs)*. The four themes capture how and under which conditions staff trauma in the form of the 4Fs is caused, fueled and reinforced within emergency care settings. Clinicians described operating in persistent states of hyperarousal consistent with the 4Fs underpinned by the anxiety of being physically or verbally attacked. The 4Fs offer ways to respond to such attacks through preparedness to fight, to escape the situation, to shut down, or to manoeuvre a path to safety through compromise and distraction.

Clinicians in the ED describe heightened awareness of potential threats, often primed for violence before it occurs. Staff report hypervigilance, hyperarousal, fear, anxiety, defensiveness, emotional withdrawal, and efforts to placate or fast-track potentially violent patients — a state of arousal that can impair cognitive capacity to respond effectively even before a behavioural issue arises. Staff in our study describe a distressing loss of agency, burnout, erosion of professional identity, acute moral injury, and substantial impacts on their personal lives, linked to persistent psychological stress indicative of significant cumulative occupational harm (47, 52, 63–66). Prolonged hyperarousal contributes to irritability, moral distress, and exhaustion (67); flight responses evolve into disengagement, burnout, and workforce attrition (62); and freeze responses reflect cumulative decision fatigue and emotional shutdown (48).

The fawn material implies a testable hypothesis: if aggressive patients are routinely prioritised to de-escalate them, triage may be reinforcing the very behaviour it aims to contain — a pattern of intermittent reinforcement, where aggression is rewarded with faster attention and relaxed boundaries. The fawn quotations in Patient Complexity state this almost explicitly. Fawning responses, while often reasonable and well-intentioned, risk eroding professional boundaries; because this erosion tends to track with patient aggression, it can inadvertently reinforce the behaviour clinical staff are trying to avoid (68).

Critically, maladaptive behaviour can occur over time driven by system conditions that normalise repeated threat exposure while constraining clinicians’ capacity to respond differently. So, maladaptive survival responses were reinforced rather than resolved, contributing to cumulative occupational harm. Staff describe acts of aggression towards patients, a lack of trust in the safety and predictability of the ED environment, reduced confidence in the team, and cynicism towards the organisation. These experiences erode team cohesion, increase risk to staff and patients and exacerbate the cycle of workforce loss, a trend seen internationally in the United Kingdom and the United States (4, 5).

Varied strategies have been implemented to address WPV, such as environmental modifications, increased security, surveillance, and dedicated mental health spaces (12, 19). Staff training in de-escalation and restraint procedures (52, 63, 64) is frequently recommended as is the promotion of clinician resilience (68). However, when implemented in isolation from broader organisational and structural reform (69) these approaches are perceived as disconnected from everyday clinical realities. High staff turnover and increasing reliance on casual and locum staff further undermine the continuity of training and organisational learning (26, 56).

Increasingly, literature underscores the pivotal role of organisational leadership in addressing workplace safety culture, ensuring adequate resourcing, and supporting staff wellbeing (12, 28, 55). However, outcome measures such as psychological safety and job-related affect remain underrepresented in the evaluation of violence prevention interventions (43). Without focus on these workforce indicators, organisations risk misinterpreting reduced incident reporting as improved safety.

Given the seriousness of WPV in EDs, there is a stark disparity in how hospitals prepare clinicians for medical versus behavioural emergencies. ED staff are extensively trained, rehearsed, and assessed in managing cardiac arrest (Code Blue) and major trauma, with well-defined team roles and structured algorithms. Management of behavioural emergencies (Code Black), by contrast, lacks equivalent standardisation, leaving staff to respond from a dysregulated state — fight, flight, freeze, or fawn — rather than trained protocol. This is a patient safety issue, not only a staff wellbeing one: a frozen or placating clinician is not delivering good care, and the patient is often delirious, intoxicated, or in crisis and least able to compensate. The quotation provided by a doctor (see Safety Systems Interface) which states that sedation is chosen because it is quicker, illustrates this — the intervention reflects the clinician’s dysregulation, not clinical judgement. This aligns with literature on patients’ experience of restraint (37) which finds coercive care more distressing particularly when rushed or inconsistent — the conditions an unsupported, dysregulated response produces. Staff dysregulation and patient harm during restraint are two sides of the same problem.

The integration of the qualitative and quantitative data revealed a significant gap between individual confidence and system-level enactment. Many survey respondents reported confidence in discrete Code Black tasks, yet qualitative accounts describe inconsistent protocol adherence, variable training, role ambiguity, and difficulty coordinating clinical and security responses — indicating that individual confidence does not translate into a coordinated, predictable team response. This points to a need for a formalised, policy-driven approach, with clear protocols and regular training to convert individual confidence into reliable team-level performance. Treating behavioural and medical emergencies unequally depletes staff resources and resilience, as clinicians repeatedly cycle through the 4F responses during activations that occur without organisational protection. Behavioural emergencies should be elevated to the same level of clinical concern as other high-risk events (48, 62, 67) — which, in practice, means mandatory competency requirements and corresponding prerequisite training.

Aligning behavioural emergency training with the standardisation applied to medical emergencies (e.g. Code Blue) has a direct evidence base. The stress inoculation and stress exposure training literature addresses how graded, rehearsed exposure to stressors alters performance under threat, helping individuals maintain effective functioning rather than defaulting to fight, flight, freeze, or fawn (70, 71). A recent systematic review of de-escalation curricula in health professions education reinforces this: interventions with the strongest evidence combined didactic teaching with role-play or simulation, rather than either alone (72). This would lead to training that is graded, rehearsed, and simulation-based, mirroring the existing model for Code Blue.

Staff require structured education on the causes and consequences of behavioural emergencies for patients and clinicians alike, alongside training in real-time management and post-incident processes such as debriefing. A cohesive training framework should be embedded both at the tertiary level and as a prerequisite for ED onboarding, supported by regular in-service education. Regular role rotation to limit time in high-stress roles, and embedded access to psychological support, must become standard across all EDs (56).

Future evaluations should use a multilevel outcome set — spanning implementation, staff, service, and patient domains — that extends beyond incident counts to assess whether interventions were adopted, delivered as intended, sustained, and effective, including reach, acceptability, feasibility, fidelity, cost, and sustainability (73).

Staff wellbeing outcomes should capture perceived safety, psychological safety, burnout, post-traumatic stress, and job satisfaction, ideally via validated instruments, supplemented by injury rates, lost workdays, and workers’ compensation claims. Confidence in leadership responsiveness and willingness to report violence would similarly indicate the strength of the underlying safety culture.

Behavioural-emergency management outcomes might track time from recognition of escalating behaviour to Code Black activation, use of de-escalation before restraint or sedation, and adherence to defined clinical and security roles and protocols. Training could be judged against completion rates, demonstrated competency, and staff confidence in managing behavioural emergencies, while incident-review and debrief completion rates offer a marker of organisational follow-through. Workforce outcomes should include turnover, retention, and leave uptake while patient restraint rate and duration, adverse events, and patient-reported experience would provide data from the patient side.

Our findings indicate that violence or threats of violence in the ED should be conceived of as complex clinical events that have both short-term and longer-term ramifications- including the enduring physical and psychological consequences for those who work there. Behavioural emergencies warrant the same level of preparatory and prerequisite training for ED clinicians as clinical emergencies. Genuine cultural change—within both hospital administration and frontline teams—is essential to ensure the seriousness of this issue is fully understood and appropriately addressed.

At the core of this discussion lies the disjunction between organisational rhetoric endorsing staff wellbeing and actual leadership accountability for implementing meaningful, system-level change. Without consistent action to reduce violence and aggression in the ED—and to adequately prepare and support staff before, during, and after behavioural emergencies—these commitments remain largely aspirational.

### 4.1 Limitations

Since all participants were recruited from a single Australian health district, transferability is a limitation. Many findings converge with international emergency services—e.g., COVID-19’s impact, rising ED attendances, overcrowding, access block, and staff shortages—though Australia has unique diverging issues, such as declining GP bulk billing, mental health care pathways, and NSW’s psychiatrist shortage.

Recall bias is a further limitation: staff self-reported violence and aggression experiences from any point they remembered and chose to discuss, likely favouring more salient incidents. Findings should be interpreted accordingly.

**Table 1** counts also warrant caution: codeable material varied by source (a 90-minute interview yields far more than a brief survey response), and distribution across INT2019, FG2021, SUR2023 and INT2023 is not reported. Counts reflect relative prominence within the dataset, not generalisable proportions, and should not be read as evidence of how common particular responses are in ED practice generally.

A further limitation is that data were collected across pre-intervention (2019) and post-intervention (2021 and 2023) phases. This approach supported identification of common trauma-response patterns across this programme of research. It may have obscured changes associated with implementation of the restructured Code Black response. The study was not designed as a longitudinal intervention evaluation. Future research would be needed to examine changes in staff experiences and 4F responses over time following the introduction of the interventions to manage behavioural emergencies.

## 5 Conclusion

Violence and aggression in emergency departments are often linked to predictable and persistent consequences of sustained system pressure, access block, and insufficient organisational responses. Within this system clinicians respond to threatening incidents with thoughts, emotions and behaviours that can be described under the 4Fs – fight, flight, freeze and fawn. Inadequate implementation of existing strategies to manage incidents of WPV leaves clinicians underprepared and operating in chronic states of stress, resulting in cumulative occupational harm, including burnout, moral injury, and loss of trust in leadership. Critically, this study has identified the lack of formal recognition of behavioural emergencies as legitimate clinical events, in contrast to the structured preparation afforded to medical emergencies. Addressing this requires elevating behavioural emergencies to the same clinical priority, supported by standardised training, clear roles, and consistent post-incident reporting and support. Without system-level reform and leadership accountability, emergency departments continue to jeopardise the health and wellbeing of clinicians and patients, workforce sustainability and safety of care.

## Data Availability

All de-identified raw data supporting the conclusions of this article will be made available by the authors, without undue reservation.

## Footnotes

1 Counts reflect the number of coded instances, not incidents or participants, and are not mutually exclusive across codes; see Method for further detail.

2 Two instances described extended bouts of fight behaviour that oscillated with strategic withdrawal; both were coded under Fight because the situation was ultimately resolved through sedation and restraint.
